# Analysis of influencing factors of concurrent primary liver cancer in hepatitis B patients and construction of column chart prediction model

**DOI:** 10.1017/S0950268825100538

**Published:** 2025-09-16

**Authors:** Qunmei Cao, Yilin Zhou, Changlong Wen, Qinglan Li

**Affiliations:** Department of Infectious Disease, Ganzhou People’s Hospital, Ganzhou, China

**Keywords:** column chart, hepatitis B, influencing factors, primary liver cancer, prediction model

## Abstract

A predictive column chart was developed to assess the risk of primary liver cancer (PLC) in hepatitis B patients. Data from 107 PLC patients and 107 controls were used as the training set, with 92 patients as the validation set. An additional 446 patients from other hospitals, including 15 with PLC, formed the external validation group. Multivariate logistic regression identified gender, BMI, alcohol consumption, diabetes, family history of liver cancer, cirrhosis, and HBV DNA load as independent risk factors. The model showed strong discrimination with AUCs of 0.882 and 0.859 in the training and validation sets, respectively, and good calibration (Hosmer–Lemeshow χ² = 2.648, P = 0.954; χ² = 4.117, P = 0.846). Decision curve analysis (DCA) confirmed clinical benefit within a risk threshold of 0.07–0.95. In the external validation group, the model maintained discrimination (AUC = 0.863) and calibration (Hosmer–Lemeshow χ² = 7.999, P = 0.434), with DCA showing net benefit across 0.14–0.95. These results indicate the column chart is a reliable tool for PLC risk prediction in hepatitis B patients.

## Introduction

Currently, a significant portion of primary liver cancer cases is caused by HBV. If early large-scale intervention measures can be implemented, liver cancer may become the second cancer, following cervical cancer, to be effectively controlled globally [1]. Therefore, it is crucial in clinical practice to assess the risk of primary liver cancer in HBV patients early on, in order to guide patients through validated interventions such as vaccination, reduce the incidence of liver cancer, and improve their prognosis. Many studies have analysed the influencing factors of primary liver cancer, but there is still a need to further explore systematic liver cancer risk-prediction tools [2–4]. Various risk scores have already been developed to predict the risk of liver cancer. The PAGE-B risk score is used to predict liver cancer risk in white patients receiving antiviral therapy, while the REACH-B, GAG-HCC, and CU-HCC risk scores are applicable to untreated Asian populations [5]. These risk scores use factors such as age, gender, HBV DNA viral load, and liver cirrhosis to predict liver cancer risk. However, these scores lack the ability to explain the impact of comorbidities and other factors such as diabetes, hypertension, and obesity on liver cancer. Additionally, the complexity of these scores makes them difficult to use in clinical practice, limiting their convenience. This study analyses the influencing factors of primary liver cancer in HBV patients and incorporates some of the aforementioned factors to construct a nomogram prediction model. By combining this with existing risk scores, the prediction of liver cancer risk may be further improved.

## Subjects and methods

### Study subjects

A retrospective selection of 153 patients with HBV complicated by primary liver cancer and 153 patients with HBV without primary liver cancer, treated in our hospital between January 2022 and August 2024, was used as the study subjects. The patients were randomly assigned into a training set and a validation set in a 7:3 ratio (This allows for a relatively larger validation dataset, enabling a better assessment of the model’s generalization performance. Moreover, it makes the validation results more representative, especially when the dataset is large, as this ratio can effectively evaluate the model’s performance across different scenarios.). The validation set included 107 patients with HBV complicated by primary liver cancer (complicated group) and 107 patients with HBV without primary liver cancer (non-complicated group), with a total of 92 patients in the validation set (46 patients each for the complicated and non-complicated groups). The external validation group comprised 446 hepatitis B patients from other hospitals, including 15 with primary liver cancer.

**Figure 1.**
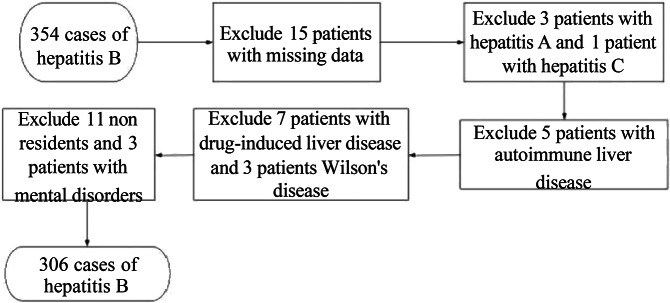
Case collection flowchart.

Inclusion criteria: (i) meet the diagnostic criteria for HBV and primary liver cancer [6, 7]; (ii) mentally stable, capable of normal communication; (iii) the study has been approved by the Medical Ethics Committee; and (iv) local permanent residents who have lived locally for more than 3 years. Exclusion criteria: (i) patients with drug-induced liver disease, Wilson’s disease, or autoimmune liver disease; (ii) patients with primary tumours of other systems; (iii) missing clinical test data; and (iv) patients with hepatitis caused by hepatitis A or C virus. The flowchart of case collection is shown in Figure 1.

**Figure 2.**
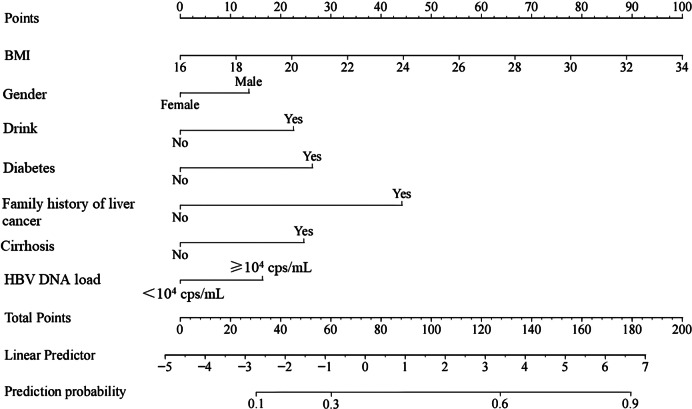
Nomogram prediction model for HBV patients complicated with primary liver cancer.

### Collection of clinical data

The clinical data collected from patients included: gender, age, body mass index (BMI), smoking, drinking, hypertension, hyperlipidaemia, diabetes, monthly income, education level, family history of liver cancer, alcoholic fatty liver disease, liver cirrhosis, dietary habits, HBV DNA viral load, and antiviral treatment.

### Statistical analysis

Statistical analysis was performed using IBM-SPSS 25.0 software and R software (version R4.3.3). Measurement data [Mean ± standard deviation/(±)] were analysed using an independent sample t-test, and categorical data [Number of cases (percentage)/n (%)] were compared using the *χ^2^* test. Multivariate logistic regression analysis was used to screen independent risk factors for primary liver cancer in HBV patients. Variables with P < 0.05 in the multivariate logistic regression analysis were included as predictive factors, and a nomogram prediction model was constructed using the RMS package in R software. ROC curves and calibration curves were used to analyse the nomogram’s ability to predict primary liver cancer in HBV patients. Decision curve analysis was performed to reflect the clinical net benefit brought by the nomogram to patients. A two-sided P-value <0.05 was considered statistically significant.

## Results

### Comparison of clinical data between the training set and validation set

There were no statistically significant differences (P > 0.05) in gender, age, BMI, smoking, drinking, hypertension, hyperlipidaemia, diabetes, monthly income, education level, family history of liver cancer, alcoholic fatty liver disease, liver cirrhosis, dietary habits, HBV DNA viral load, and antiviral treatment between the training set and the validation set. See Table 1.

**Table 1.** Comparison of clinical data between the training set and validation set [*n*(%)/(±)]

Index	Training set (*n* = 214)	Verification set (*n* = 92)	*χ^2^/t*	*P*
Gender [*n*(%)]			0.812	0.368
Female	70 (32.71)	35 (38.04)		
Male	144 (67.29)	57 (61.96)		
Age (years)	51.31 ± 9.48	52.95 ± 9.62	1.381	0.168
BMI (kg/m^2^)	22.83 ± 2.57	22.25 ± 2.68	1.787	0.075
Smoke [*n*(%)]	104 (48.60)	55 (59.78)	3.224	0.073
Drink [*n*(%)]	57 (26.64)	18 (19.57)	1.738	0.187
Hypertension [*n*(%)]	37 (17.29)	12 (13.04)	0.863	0.353
Hyperlipidaemia [*n*(%)]	20 (9.35)	15 (16.30)	3.076	0.079
Diabetes [*n*(%)]	35 (16.36)	10 (10.87)	1.544	0.214
Monthly income [*n*(%)]			1.322	0.250
<3,500 yuan	60 (28.04)	20 (21.74)		
≥3,500 yuan	154 (71.96)	72 (78.26)		
Educational level [*n*(%)]			1.285	0.526
Primary school	36 (16.82)	12 (13.04)		
Junior high school	116 (54.21)	56 (60.87)		
High school and above	62 (28.97)	24 (26.09)		
Family history of liver cancer [*n*(%)]	23 (10.75)	7 (7.61)	0.717	0.397
Alcoholic fatty liver [*n*(%)]	20 (9.35)	12 (13.04)	0.939	0.332
Cirrhosis [*n*(%)]	82 (38.32)	27 (29.35)	2.257	0.133
Eating habits [*n*(%)]			2.488	0.115
Good	150 (70.09)	56 (60.87)		
Poor	64 (29.91)	36 (39.13)		
HBV DNA load [*n*(%)]			2.587	0.108
<10^4^ cps/mL	156 (72.90)	75 (81.52)		
≥10^4^ cps/mL	58 (27.10)	17 (18.48)		
Antiviral therapy [*n*(%)]			1.171	0.279
Yes	144 (67.29)	56 (60.87)		
No	70 (32.71)	36 (39.13)		

### Univariate analysis of HBV patients complicated with primary liver cancer in the training set

There were no statistically significant differences (P > 0.05) in age, smoking, hypertension, hyperlipidaemia, monthly income, education level, alcoholic fatty liver disease, dietary habits, and antiviral treatment between the non-complicated group and the complicated group in the training set. However, the proportions of males, alcohol consumption, diabetes, family history of liver cancer, liver cirrhosis, HBV DNA viral load ≥10^4^ cps/mL, and BMI were higher in the complicated group compared to the non-complicated group (P < 0.05). See Table 2.

**Table 2.** Univariate analysis of HBV patients complicated with primary liver cancer in the training set[*n*(%)/(±)]

Index	Not concurrent group (*n* = 107)	Concurrent group (*n* = 107)	*χ^2^/t*	*P*
Gender [*n*(%)]			16.644	0.000
Female	49 (45.79)	21 (19.63)		
Male	58 (54.21)	86 (80.37)		
Age (years)	50.24 ± 9.17	52.38 ± 9.76	1.653	0.100
BMI (kg/m^2^)	21.55 ± 2.36	24.10 ± 2.78	7.233	0.000
Smoke [*n*(%)]	45 (42.06)	59 (55.14)	3.666	0.056
Drink [*n*(%)]	15 (14.02)	42 (39.25)	17.433	0.000
Hypertension [*n*(%)]	15 (14.02)	22 (20.56)	1.601	0.206
Hyperlipidaemia [*n*(%)]	8 (7.48)	12 (11.21)	0.882	0.348
Diabetes [*n*(%)]	7 (6.54)	28 (26.17)	15.064	0.000
Monthly income [*n*(%)]			0.371	0.543
<3,500 yuan	32 (29.91)	28 (26.17)		
≥3,500 yuan	75 (70.09)	79 (73.83)		
Educational level [*n*(%)]			1.293	0.524
Primary school	15 (14.02)	21 (19.63)		
Junior high school	59 (55.14)	57 (53.27)		
High school and above	33 (30.84)	29 (27.10)		
Family history of liver cancer [*n*(%)]	3 (2.80)	20 (18.69)	14.078	0.000
Alcoholic fatty liver [*n*(%)]	12 (11.21)	8 (7.48)	0.882	0.348
Cirrhosis [*n*(%)]	25 (23.36)	57 (53.27)	20.245	0.000
Eating habits [*n*(%)]			1.427	0.232
Good	79 (73.83)	71 (66.36)		
Poor	28 (26.17)	36 (33.64)		
HBV DNA load [*n*(%)]			18.543	0.000
<10^4^ cps/mL	92 (85.98)	64 (59.81)		
≥10^4^ cps/mL	15 (14.02)	43 (40.19)		
Antiviral therapy [*n*(%)]			3.057	0.080
Yes	78 (72.90)	66 (61.68)		
No	29 (27.10)	41 (38.32)		

### Logistic regression analysis of factors influencing HBV patients complicated with primary liver cancer

Variables with statistically significant differences in the univariate analysis from Table 2 were included in the logistic regression analysis. The variable assignments are shown in Table 3. The results of multivariate analysis are shown in Table 4, where gender, BMI, alcohol consumption, diabetes, family history of liver cancer, liver cirrhosis, and HBV DNA viral load were all found to be influencing factors for HBV patients complicated with primary liver cancer (P < 0.05).

**Table 3.** Variable assignment table

Influence factor	Assignments
Gender	Male = 1, Female = 0
BMI	Continuous variable
Drink	Yes = 1, No = 0
Diabetes	Yes = 1, No = 0
Family history of liver cancer	Yes = 1, No = 0
Cirrhosis	Yes = 1, No = 0
HBV DNA load	≥10^4^ cps/mL = 1, <10^4^ cps/mL = 0
Concurrent primary liver cancer	Yes = 1, No = 0

**Table 4.** Multivariate logistic regression analysis

Influence factor	*β*	*SE*	*Waldχ^2^*	*OR*	*95%CI*	*P*
Gender	0.857	0.390	4.835	2.356	1.098 ~ 5.059	0.028
BMI	0.349	0.082	17.982	1.417	1.206 ~ 1.665	0.000
Drink	1.415	0.440	10.338	4.115	1.737 ~ 9.749	0.001
Diabetes	1.649	0.590	7.826	5.204	1.639 ~ 16.528	0.005
Family history of liver cancer	2.769	0.794	12.158	15.948	3.362 ~ 75.638	0.000
Cirrhosis	1.543	0.393	15.429	4.680	2.167 ~ 10.110	0.000
HBV DNA load	1.026	0.432	5.642	2.791	1.197 ~ 6.510	0.018
Constant	−10.198	1.971	26.762	0.000	-	0.000

### Construction of the nomogram for predicting primary liver cancer in HBV patients

Based on the variables with statistically significant differences from the analysis results in Table 4, a nomogram was constructed using R software to predict primary liver cancer in HBV patients (see Figure 2). Each variable was assigned a score from 0 to 100 proportionally, with upward vertical lines used to determine the score for each variable and downward vertical lines used to determine the total score’s corresponding predicted probability, which represents the risk of primary liver cancer in HBV patients.

### Internal validation of the prediction model for primary liver cancer in HBV patients

**Figure 3.**
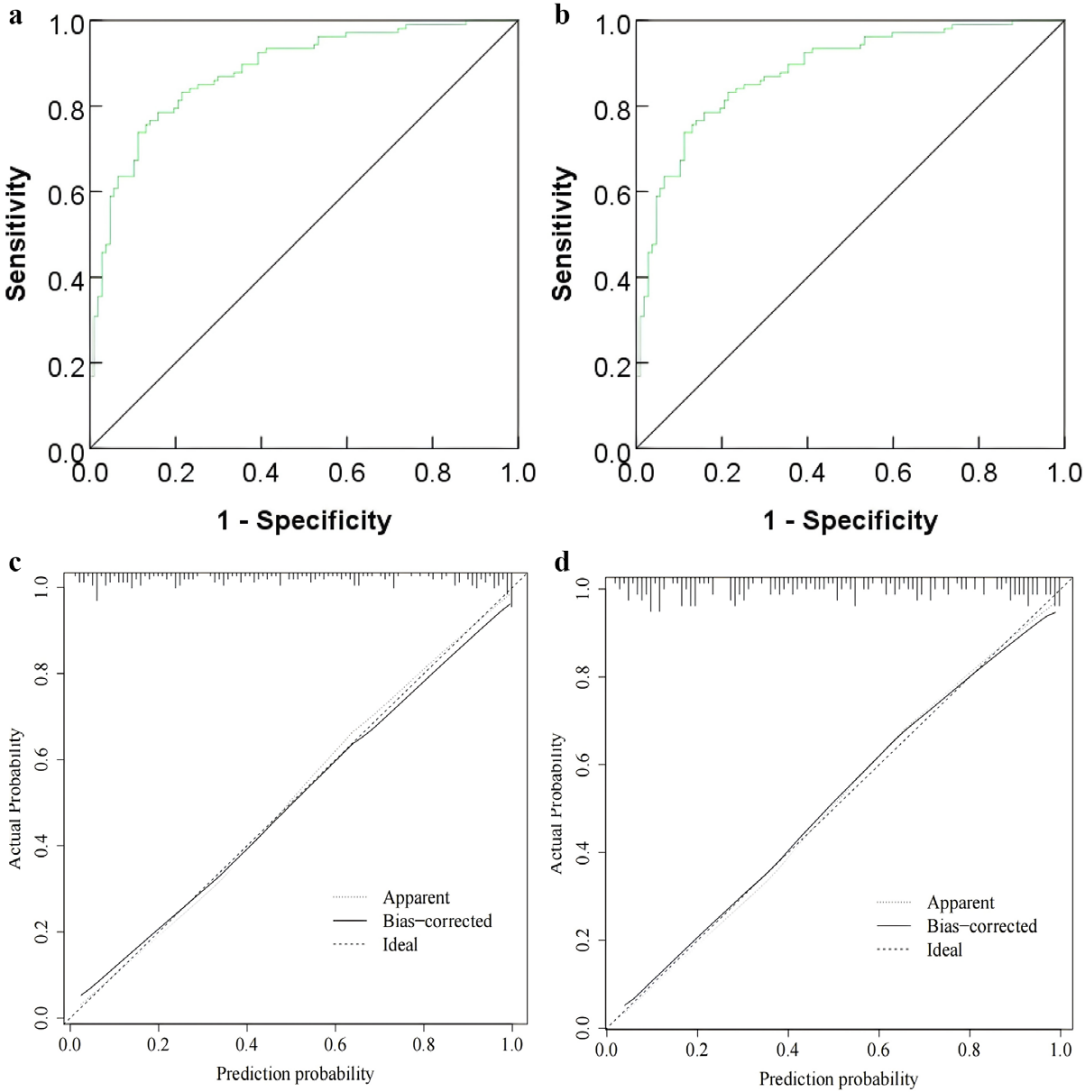
ROC curves (a, b) and calibration curves (c, d) of the prediction model for primary liver cancer in HBV patients in the training set and validation set.

The ROC curve results for the training set (Figure 3a) and the validation set (Figure 3b) showed that the AUCs were 0.882 (95%CI: 0.837 ~ 0.926) and 0.859 (95%CI: 0.810 ~ 0.909), respectively, indicating that the nomogram prediction model had high discriminative power.

**Figure 4.**
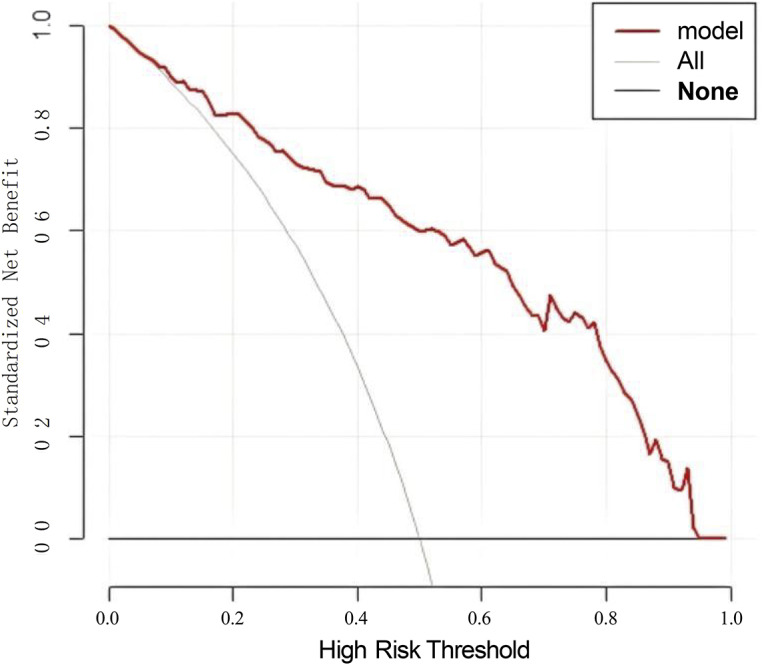
Decision curve analysis diagram.

The calibration curve results for the training set (Figure 3c) and the validation set (Figure 3d) showed that the Hosmer–Lemeshow test yielded *χ^2^* = 2.648, 4.117, *P* = 0.954, 0.846, respectively, indicating that the nomogram prediction model matched the results in both cohorts, with a good degree of agreement between the model predictions and the actual outcomes.

### Analysis of the clinical application value of the nomogram model

The decision curve analysis results showed that in the high-risk threshold probability range of 0.07 ~ 0.95, the nomogram model’s line was above both the ‘All’ line and the ‘None’ line, indicating a good net positive benefit and high clinical value. The ‘All’ line represents the net benefit when all hepatitis B patients receive treatment, while the ‘None’ line represents the net benefit when no hepatitis B patients receive treatment (see Figure 4).

### External validation of the prediction model for primary liver cancer in HBV patients

**Figure 5.**
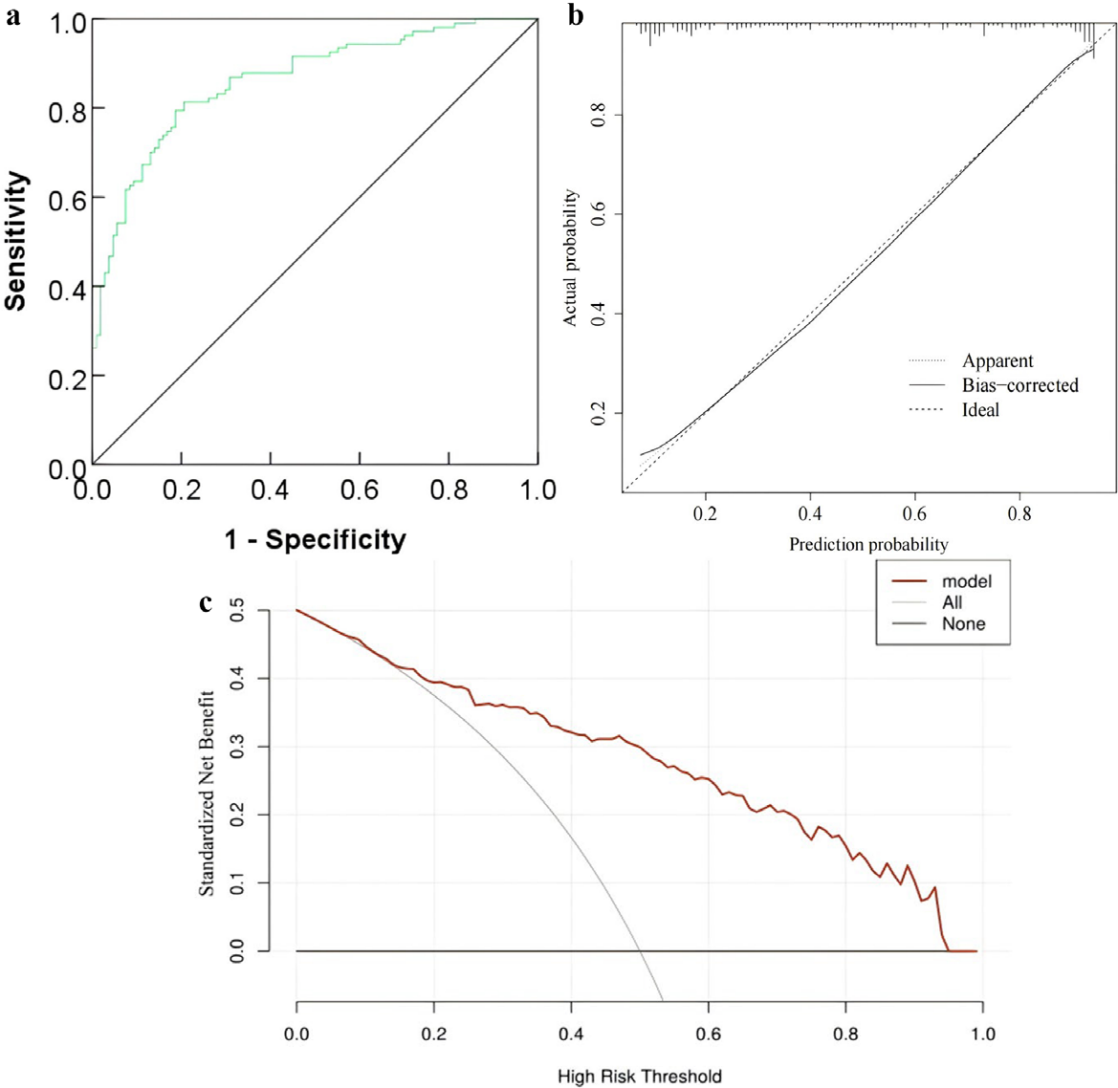
ROC curve (a), calibration curve (b), and decision curve analysis (b) of the prediction model for primary liver cancer in HBV patients in the external validation group.

The ROC curve for external validation showed an AUC of 0.863 (95% CI: 0.814–0.911), indicating good discriminatory ability of the nomogram model. The calibration curve demonstrated good agreement between predicted and actual outcomes, with a Hosmer–Lemeshow test result of χ^2^ = 7.999 and P = 0.434. Decision curve analysis showed that within a high-risk threshold probability range of 0.14 to 0.95, the nomogram curve lay above the ‘All’ and ‘None’ lines, suggesting favourable net clinical benefit and high clinical utility (Figure 5).

## Discussion

The HBV virus is a DNA-based virus, belonging to the family of hepatotropic DNA viruses. It replicates extensively within liver cells and interacts with various cellular proteins, causing hepatitis B in infected individuals and increasing the risk of liver cancer [8]. Prevention is the most effective method for controlling liver cancer. Liver cancer prevention is divided into three levels: primary prevention involves vaccination for the general population starting from birth; secondary prevention involves the use of antiviral drugs for high-risk individuals with chronic HBV infection; and tertiary prevention also uses antiviral drugs to prevent recurrence in high-risk individuals with chronic HBV infection [9]. Therefore, it is necessary to accurately assess high-risk patients with primary liver cancer among HBV patients based on relevant influencing factors to guide clinical treatment.

Multivariate regression analysis in this study revealed that gender, BMI, alcohol consumption, diabetes, family history of liver cancer, liver cirrhosis, and HBV DNA viral load are important risk factors for HBV patients complicated with primary liver cancer. (1) Gender and Alcohol Consumption: The study by Xu et al. [10] showed that aflatoxin exposure increases the risk of hepatocellular carcinoma in HBV patients, with male patients exhibiting higher expression levels of genes related to aflatoxin metabolism than female patients. Wang et al. [11] found that androgens enhance the genotoxicity and inflammation induced by aflatoxin in male HBV patients, which may explain the higher risk of liver cancer in male patients. Additionally, alcohol-related liver disease is a major cause of chronic liver disease worldwide, and it may contribute to metabolic dysfunction in patients, thereby playing a role in the progression of liver cancer [12]. (2) BMI: Fan et al. [13] found that central obesity is a risk factor for hepatocellular carcinoma in Asian patients with chronic HBV infection. Obesity, characterized by excessive fat accumulation in the body, is generally indicated by a high BMI. The liver, being the main organ for fat storage, secretes pro-inflammatory cytokines, which act as carcinogenic signalling mediators in the progression of liver cancer [14]. (3) Diabetes: Yip et al. [15] suggested that whether chronic HBV patients also have diabetes affects the accuracy of multiple liver cancer risk scores. This may indicate that the liver cancer risk profiles differ between chronic HBV patients with and without diabetes, and existing scores cannot strictly distinguish between them. Furthermore, Campbell et al. [16] found that diabetes is an independent risk factor for liver cirrhosis and hepatocellular carcinoma in chronic HBV patients. Previous observational studies have shown that the global burden of primary liver cancer is exacerbated by the coexistence of type 2 diabetes, with the disease burden consistently higher in males across all age groups [17]. It is hypothesized that diabetes, closely associated with insulin resistance and hyperglycaemia, may lead to dysregulated intracellular signalling pathways and promote the development of liver cancer. (4) Family History of Liver Cancer and HBV DNA Viral Load: Previous studies have found significant familial clustering of primary liver cancer, mainly influenced by genetic factors. Relevant oncogenes can integrate into liver cells through the replication of HBV DNA, leading to the occurrence and progression of liver cancer [18]. A higher HBV DNA viral load represents a higher level of viral replication in the patient, increasing the probability of primary liver cancer. (5) Liver Cirrhosis: Huang et al. [19] found that liver cirrhosis is a significant risk factor for primary liver cancer. Cirrhosis induced by HBV increases the risk of liver tissue fibrosis, leading to abnormal immune system responses, which in turn cause elevated expression of tumour factors and decreased expression of anti-tumour factors, ultimately resulting in primary liver cancer [20].

In recent years, nomogram models have gained widespread popularity due to their convenient modelling approach and ability to integrate multiple variables. If the AUC of a nomogram model exceeds 0.7, the model is considered to have good discriminative power [21]. Wu et al. [22] found that the REAL-B risk score had high discriminative power in predicting hepatocellular carcinoma in chronic HBV patients, with AUCs of 0.76 (3 years) and 0.75 (5 years) through internal and external validation. In this study, the performance of the nomogram model was evaluated using ROC curves and calibration curves. The results showed that the AUCs of the model in the training set and validation set were 0.882 and 0.859, respectively, both above 0.7 and higher than those in the aforementioned studies. Additionally, the calibration curves followed the reference lines. Therefore, we preliminarily conclude that the nomogram model constructed in this study has good discriminative and calibration capabilities, making it useful for predicting liver cancer. Decision curve analysis in this study showed that the nomogram could yield net benefits within most reasonable high-risk threshold probability ranges, suggesting that it could help clinicians promptly detect disease progression in patients and take effective intervention measures to improve their quality of life. Further external validation using cases from other hospitals demonstrated that the model maintained high discriminative ability, with an AUC of 0.863 on the ROC curve, consistent with the internal validation results from our hospital. The calibration curve also showed good agreement between predicted and observed probabilities, indicating high calibration accuracy. Decision curve analysis confirmed that the nomogram continued to provide a high net benefit in external hospital settings, supporting its value in clinical decision-making.

However, the results of this study may have the following limitations and shortcomings. Firstly, this was a retrospective study, so inaccuracies may arise due to the small data size, and there are inevitable confounding biases. Prospective clinical studies are needed to verify the current results. Secondly, some potential influencing factors (e.g., genetic status, biomarkers) are still unclear, and including such important factors may further improve the nomogram’s effectiveness. Thirdly, due to limitations in the timeframe of case inclusion, the nomogram has not yet reached an ideal state, with AUCs of only 0.882 and 0.859, leaving room for improvement, which requires further refinement. Fourthly, the generalizability of the model constructed in this study requires further validation. The current dataset has no missing values and includes multiple exclusion criteria, which may affect the model’s applicability to other datasets. Fifth, the validation sample size is relatively small, and each variable in the multivariate analysis does not meet the principle of at least 10 events per variable, potentially leading to low statistical power of the model.

## Conclusion

In conclusion, this study constructed a nomogram for predicting primary liver cancer in HBV patients based on an analysis of influencing factors. The nomogram not only includes baseline information such as gender, BMI, alcohol consumption, diabetes, family history of liver cancer, liver cirrhosis, and HBV DNA viral load but also greatly improves the sensitivity and specificity of the model. If the nomogram can be validated in an external dataset that includes a broader population representing real-world clinical data, it can be used to help clinicians identify high-risk individuals and implement targeted screening and personalized treatment.

## Data Availability

The datasets used and/or analysed during the present study are available from the corresponding author on reasonable request.

## References

[1] Shi J-F, Cao M, Wang Y, et al. (2021) Is it possible to halve the incidence of liver cancer in China by 2050? International Journal of Cancer 148(5), 1051–1065. 10.1002/ijc.33313. 32997794

[2] Cao M, Li H, Sun D, et al. (2022) Assessment of the compliance, influencing factors, and yielding results of liver cancer screening in a high-risk population: A cross-sectional study. Cancer 128(20), 3653–3662. 10.1002/cncr.34418. 35996957

[3] Zhang H and Liu J (2024) Lifestyle factors, glycemic traits, and lipoprotein traits and risk of liver cancer: A Mendelian randomization analysis. Scientific Reports 14(1), 8502. 10.1038/s41598-024-59211-3. 38605235 PMC11009263

[4] Zhao J, Bai D, Qi L, et al. (2023) The flavonoid GL-V9 alleviates liver fibrosis by triggering senescence by regulating the transcription factor GATA4 in activated hepatic stellate cells. British Journal of Pharmacology 180(8), 1072–1089. 10.1111/bph.15997. 36455594

[5] Chun HS, Papatheodoridis GV, Lee M, et al. (2024) PAGE-B incorporating moderate HBV DNA levels predicts risk of HCC among patients entering into HBeAg-positive chronic hepatitis B. Journal of Hepatology 80(1), 20–30. 10.1016/j.jhep.2023.09.011. 37734683

[6] Cong W-M, Bu H, Chen J, et al. (2016) Practice guidelines for the pathological diagnosis of primary liver cancer: 2015 update. World Journal of Gastroenterology 22 (42), 9279–9287. 27895416 10.3748/wjg.v22.i42.9279 PMC5107692

[7] Terrault NA, Lok ASF, McMahon BJ, et al. (2018) Update on prevention, diagnosis, and treatment of chronic hepatitis B: AASLD 2018 hepatitis B guidance. Hepatology (Baltimore, Md.) 67(4), 1560–1599. 10.1002/hep.29800. PMC5975958 29405329

[8] Guo M, Zhao L, Jiang C, et al. (2023) Multiomics analyses reveal pathological mechanisms of HBV infection and integration in liver cancer. Journal of Medical Virology 95(8), e28980. 10.1002/jmv.28980. 37522289

[9] Singal AG, Kanwal F and Llovet JM (2023) Global trends in hepatocellular carcinoma epidemiology: Implications for screening, prevention and therapy. Nature Reviews. Clinical Oncology 20(12), 864–884. 10.1038/s41571-023-00825-3. 37884736

[10] Xu C, Cheng S, Chen K, et al. (2023) Sex differences in genomic features of hepatitis B-associated hepatocellular carcinoma with distinct antitumor immunity. Cellular and Molecular Gastroenterology and Hepatology 15(2), 327–354. 10.1016/j.jcmgh.2022.10.009. 36272708 PMC9772570

[11] Wang S-H, S-H Y and Chen P-J (2023) Androgen enhances aflatoxin-induced genotoxicity and inflammation to liver cancer in male hepatitis B patients. Cellular and Molecular Gastroenterology and Hepatology 15 (2), 507–508. 10.1016/j.jcmgh.2022.11.001. 36427539 PMC9880974

[12] Mackowiak B, Fu Y, Maccioni L, et al. (2024) Alcohol-associated liver disease. The Journal of Clinical Investigation 134(3). 10.1172/JCI176345. PMC10836812 38299591

[13] Fan R and Hou J (2021) Editorial: Central obesity is a risk factor for hepatocellular carcinoma in Asian patients with chronic hepatitis B on anti-viral therapy-authors’ reply. Alimentary Pharmacology & Therapeutics 54(5), 724–725. 10.1111/apt.16548. 34379840

[14] Sohn W, Lee HW, Lee S, et al. (2021) Obesity and the risk of primary liver cancer: A systematic review and meta-analysis. Clinical and Molecular Hepatology 27(1), 157–174. 10.3350/cmh.2020.0176. 33238333 PMC7820201

[15] Yip TC-F, Wong VW-S, Lai MS-M, et al. (2023) Diabetes mellitus impacts on the performance of hepatocellular carcinoma risk scores in chronic hepatitis B patients. Clinical Gastroenterology and Hepatology : the Official Clinical Practice Journal of the American Gastroenterological Association 21 (11). 10.1016/j.cgh.2023.02.004. 36828301

[16] Campbell C, Wang T, McNaughton AL, et al. (2021) Risk factors for the development of hepatocellular carcinoma (HCC) in chronic hepatitis B virus (HBV) infection: A systematic review and meta-analysis. Journal of Viral Hepatitis 28(3), 493–507. 10.1111/jvh.13452. 33305479 PMC8581992

[17] Xie J, Lin X, Fan X, et al. (2024) Global burden and trends of primary liver cancer attributable to comorbid type 2 diabetes mellitus among people living with hepatitis B: An observational trend study from 1990 to 2019. Journal of Epidemiology and Global Health 14(2), 398–410. 10.1007/s44197-024-00237-1. 38713342 PMC11176116

[18] Guo D, Li J, Zhao P, et al. (2024) The hepatocellular carcinoma risk in patients with HBV-related cirrhosis: A competing risk nomogram based on a 4-year retrospective cohort study. Frontiers in Oncology 14, 1398968. 10.3389/fonc.2024.1398968. 38817899 PMC11137271

[19] Huang DQ, Mathurin P, Cortez-Pinto H, et al. (2023) Global epidemiology of alcohol-associated cirrhosis and HCC: Trends, projections and risk factors. Nature Reviews. Gastroenterology & Hepatology 20(1), 37–49. 10.1038/s41575-022-00688-6. 36258033 PMC9579565

[20] Deng Y, Huang J and Wong MCS (2024) Associations of non-alcoholic fatty liver disease and cirrhosis with liver cancer in European and east Asian populations: A Mendelian randomization study. Cancer Reports (Hoboken, N.J.) 7(1), e1913. 10.1002/cnr2.1913. 37840448 PMC10809194

[21] Li F, Zheng T and Gu X (2022) Prognostic risk factor analysis and nomogram construction for primary liver cancer in elderly patients based on SEER database. BMJ Open 12(10), e051946. 10.1136/bmjopen-2021-051946. PMC9615972 36288830

[22] Wu S, Zeng N, Sun F, et al. (2021) Hepatocellular carcinoma prediction models in chronic hepatitis B: A systematic review of 14 models and external validation. Clinical Gastroenterology and Hepatology : the Official Clinical Practice Journal of the American Gastroenterological Association 19(12), 2499–2513. 10.1016/j.cgh.2021.02.040. 33667678

